# New chest compression method in infant resuscitation: Cross thumb technique

**DOI:** 10.1371/journal.pone.0271636

**Published:** 2022-08-08

**Authors:** Woochan Jeon, Jungeon Kim, Yura Ko, Jisook Lee

**Affiliations:** 1 Department of Emergency Medicine, Inje University Ilsan Paik Hospital, Goyang, Republic of Korea; 2 Department of Emergency Medicine, Ajou University, School of Medicine, Suwon, Republic of Korea; State University of New York at Buffalo, UNITED STATES

## Abstract

**Background:**

The two-thumb encircling technique (2TT) is superior to the two-finger technique (2FT) in infant cardiopulmonary resuscitation (CPR), but there are difficulties in providing ventilation as soon as possible. We modified the 2TT to the cross-thumb technique (CTT) to maintain good CPR performance at the same position as 2FT. We aimed to compare the quality of chest compression and brief hands-off times in 2FT, 2TT, and CTT by a single rescuer using an infant CPR manikin model.

**Methods:**

This study was designed as a prospective randomized controlled simulation-based study. We used the Resusci® Baby QCPR (Laerdal Medical, Stavanger, Norway) as a simulated 3-month-old infant. Ventilation was performed by the mouth-to-mouth technique using a chest compression-to-ventilation ratio of 30:2 as a single rescuer. Data on CPR quality, such as locations, rates, depth and release of chest compressions, hands-off times, and proper ventilation, were recorded using the Resusci® Baby QCPR and SkillReporter. Also, the chest compression fraction (CCF) was automatically calculated.

**Results:**

The depth of chest compression in 2FT, 2TT, and CTT were 40.0 mm (interquartile range [IQR] 39.0, 41.0), 42.0 mm (IQR 41.0, 43.0), and 42.0 mm (IQR 41.0, 43.0), respectively. The depth of chest compression in 2FT was shallower than that in the other two techniques (*P*<0.05). CCF in 2FT, 2TT, and CTT were 73.9% (IQR 72.2, 75.6), 71.2% (IQR 67.2, 72.2) and 71.3% (IQR 67.7, 74.1), respectively. CCF was higher in 2FT than in the other two techniques (*P*<0.05). Correct location in 2FT, 2TT, and CTT were 99.0% (IQR 86.0, 100.0), 100.0% (IQR 97.0, 100.0) and 100.0% (IQR 99.0, 100.0), respectively. Correct location in CTT and 2TT was higher than that in 2FT. Performing CTT, the subjective pain and fatigue score were lower than other two technique.

**Conclusion:**

A new chest compression technique, CTT was better in chest compression depth compared with 2FT and may be helpful in maintaining correct chest compression location with less pain and fatigue in infant CPR.

## Introduction

The impact of high-quality chest compressions and brief hands-off time during cardiopulmonary resuscitation (CPR) is irrefutable as it is critical for maintaining the perfusion of vital organs. High-quality chest compression and brief hands-off time help achieve recovery of spontaneous circulation and good neurologic outcomes after CPR. Therefore, recent CPR guidelines and most articles about CPR skills strongly suggest that brief hands-off time, high quality CPR performance, and minimal interruption of chest compression continued during CPR [1–4]. However, the quality of chest compressions is often poor in infant CPR. According to studies on infant CPR, the depth of chest compressions was shallow, and the no-flow time, such as hands-off time divided by cardiac arrest time, was too long [5–10].

**Fig 1:**
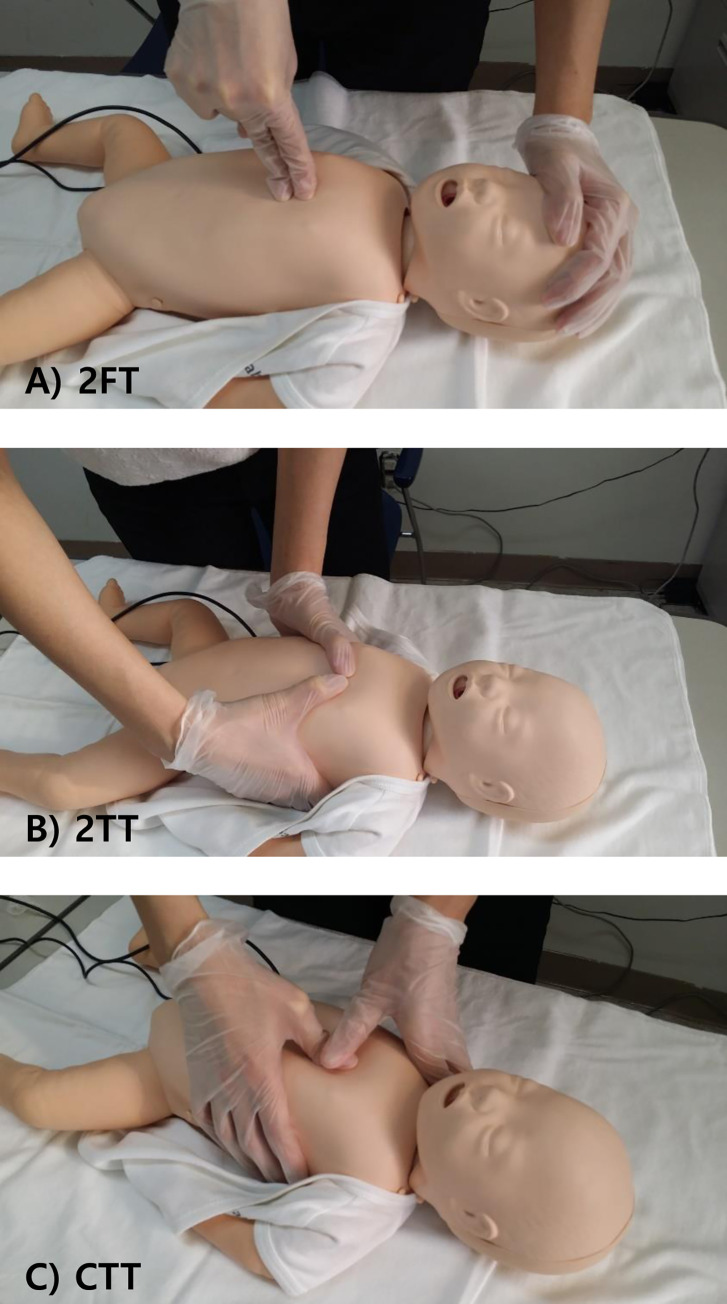
Chest compression techniques used in this study: A) Two finger technique; B) Two thumb encircling hands technique; C) Cross thumb technique.

The European Resuscitation Council and American Heart Association guidelines recommend that the lone rescuer compresses the sternum with the tip of two fingers, the so-called two-finger technique (2FT), and two or more rescuers use the two-thumb–encircling hands technique (2TT), placing both thumbs flat side by side on the lower half of the sternum just below the intermammary line with the tips pointing towards the infant’s head and spreading both hands with the fingers together to encircle the lower part of the infant’s rib cage [1, 3] (Fig 1A and 1B). Additionally, on chest compression technique in infant CPR, the 2TT is superior to the 2FT due to greater compression depth, more accurate finger placements, adaptability, and less exertion by rescuers [11]; however, the 2FT is considered superior with regard to duty cycle, chest recoil, and release force compliance [11].

**Fig 2:**
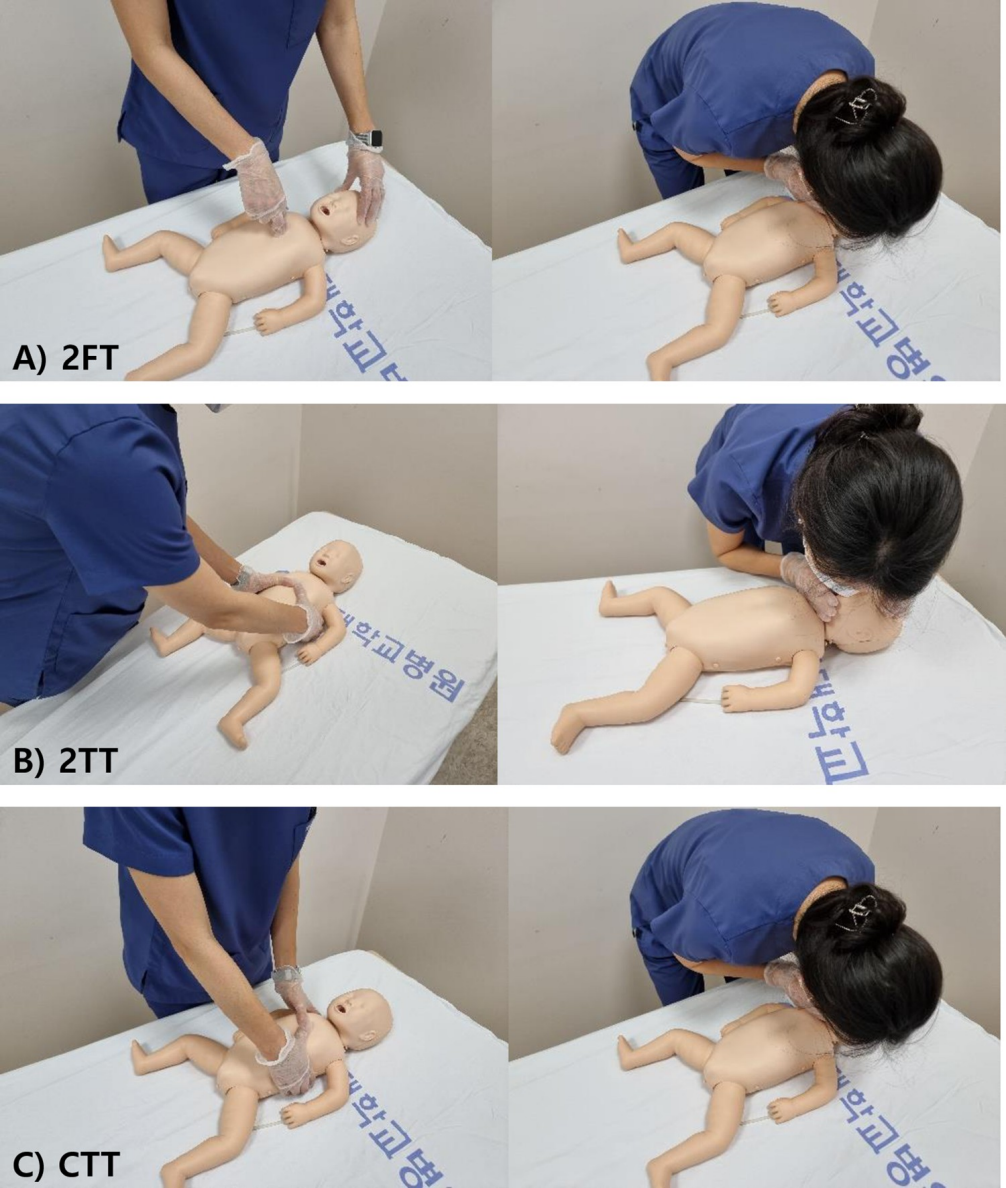
A rescuer’s location performing each chest compression technique. 2FT and CTT allow a rescuer to maintain the same position next to the patient’s chest and shoulder. With 2TT, the rescuer must perform chest compressions at the bottom of the patient. At the end of the compression cycle, the rescuer must move its position toward the patient’s head for ventilation. 2FT, two finger technique; 2TT, two thumb technique; CTT, cross-thumb technique.

The cross-thumb technique (CTT) is a novel method that modifies the 2TT to maintain high-quality chest compression and fix the location of the rescuer in relation to the infant. It is similar to 2TT, but the positions of the thumbs and hand are different. The thumb of the dominant hand is placed on the lower half of the sternum, while the dominant hand’s finger encircles the lower part of the infant’s rib cage. The thumb of the non-dominant hand is placed over the thumb of the dominant hand, and the non-dominant hand’s finger encircles the shoulder (Fig 1C). Using this technique, after breathing support without any change in the rescuer’s location, the rescuer may quickly resume chest compression at the right or left side of the patient rather than at the downside of the cardiac arrest victim. Therefore, this may promote that hands-off times are shorter than those of other techniques (Fig 2).

Furthermore, increasing the pressure by overlapping the rescuer’s thumbs and fixing the location with encircled hands suggested that high-quality chest compression could be achieved. We hypothesize that CPR performance using CTT can be more effective by using the pinch force of two thumbs while reducing the change in the rescuer’s position to minimize chest compression interruption by ventilation.

The aim of our study was to compare the quality of chest compression and brief hands-off times in 2FT, 2TT, and CTT by a single rescuer using an infant CPR manikin model.

## Materials and methods

### Study design

This study was designed as a prospective randomized controlled simulation-based study and was approved by the Institutional Review Board (ISPAIK 2019-09-06). We used the Resusci^®^ Baby QCPR (Laerdal Medical, Stavanger, Norway) as a 3-month-old infant simulating manikin. All participants were already trained in the Pediatric Basic Life Support program and were provided videos and written information about this study before participating in this study. At the beginning, the participants took oral lectures for 30 minutes. These instructions for the study comprised infant resuscitation review for high-quality CPR according to guideline, a new chest compression technique, and a study overview [1]. After instruction, all participants practiced all techniques by self-selected order to achieve high-quality chest compression and ventilation using the QCPR feedback function of simulating manikin for 10 minutes. An instructor supervised these practices and gave immediate feedback to participants during the practice time. Ventilation was performed using the mouth-to-mouth technique, using a chest compression-to-ventilation ratio of 30:2 as a single rescuer. Then, participants were randomly assigned to one of three different chest compression techniques (2FT, 2TT, and CTT) using the Research Randomized software (www.randomizer.org). Before entering the testing room, each participant selected a sealed envelope including a memo indicating the chest compression method they had been randomly selected to perform. Each participant performed five cycles of CPR using the assigned compression technique without QCPR feedback in an independent room. Data on CPR quality, such as locations, rates, depth and release of chest compressions, hands-off times, and proper ventilation, were recorded with SimPad with SkillReporter (Laerdal Medical, Stavanger, Norway). After the simulation, we surveyed the age, sex, and jobs of the participants. They answered a short questionnaire about difficulty level, fatigue, and pain scores during CPR simulation, as numeric rating scales (NRS) from 1 to 10. NRS was selected according to the participant’s experience, which meant that the lower the number, the easier it was, the less tiring it was, and the less painful it was. The higher the number, the more difficult, the more tiring and the more painful it was.

### Measurement of CPR performance

According to the American Heart Association, there are five main components of high-quality CPR: chest compression fraction (CCF), rate and depth of chest compressions, chest recoil, and ventilation [2]. CCF is the proportion of time that chest compressions are performed during a cardiac arrest. Because studies on out-of-hospital cardiac arrest have shown that low CCF is related to decreased return of spontaneous circulation (ROSC) and survival to hospital discharge, expert consensus recommended that high CCF was helpful for successful CPR in out-of-hospital cardiac arrest [12]. We recorded chest compression time and hands-off time using SkillReporter and calculated automatically CCF. Other components of high-quality CPR, such as compression rate and depth, were recorded as mean values and the percentage of success in simulation time, respectively. In accordance with the AHA and ERC guidelines, the participants were instructed to perform infant chest compression at a depth of at least 4 cm at a compression rate of 100–120 per minute. We also recorded the proportion of chest recoils in each compression and the proportion of adequate ventilation in five cycles of CPR from 0 to 100% in the SkillReporter. The chest recoil is defined as relaxation of the chest wall to its original position after each chest compression. The amount of ventilation volume measured at Resusci^®^ Baby QCPR was recorded. In consideration of the age and weight of the simulator, when the ventilation amount was between 20 ml and 40 ml, it was judged as adequate ventilation. Correct hand-position was recorded the proportion of correct location of chest compressions in five cycles of CPR using the Resusci^®^ Baby QCPR sensor. These variables were studied while being set in the SkillReporter according to the AHA guidelines, and were automatically stored and analyzed.

### Sample size

We proposed an alpha risk of 0.05 and power 0.9. The quality CPR score in our pilot data from 6 volunteers showed 88% vs. 89%. Vs 90% in the 2FT, 2TT, CTT, respectively (automatically QCPR measured by Simpad SkillReporter). Using one-way analysis of variance with 10% drop-out rate, 30 participants per a group were required.

### Statistics

Data were analyzed using SPSS v22.0 (IBM, New York, USA). Continuous variables were expressed as medians and quartiles for non-normal distribution. Nominal and categorical variables were presented as frequencies and percentages. The normal distribution of variables was confirmed using the Kolmogorov-Smirnov test. Differences in CPR parameters between the three groups were assessed using the analysis of variance (Mann-Whitney test) for the normal distribution and the analysis of variance (Kruskal-Wallis test) for the non-normal distribution. Post hoc tests were performed after correction by pairwise comparison. Statistical significance was set at *P* <0.05.

## Results

**Fig 3:**
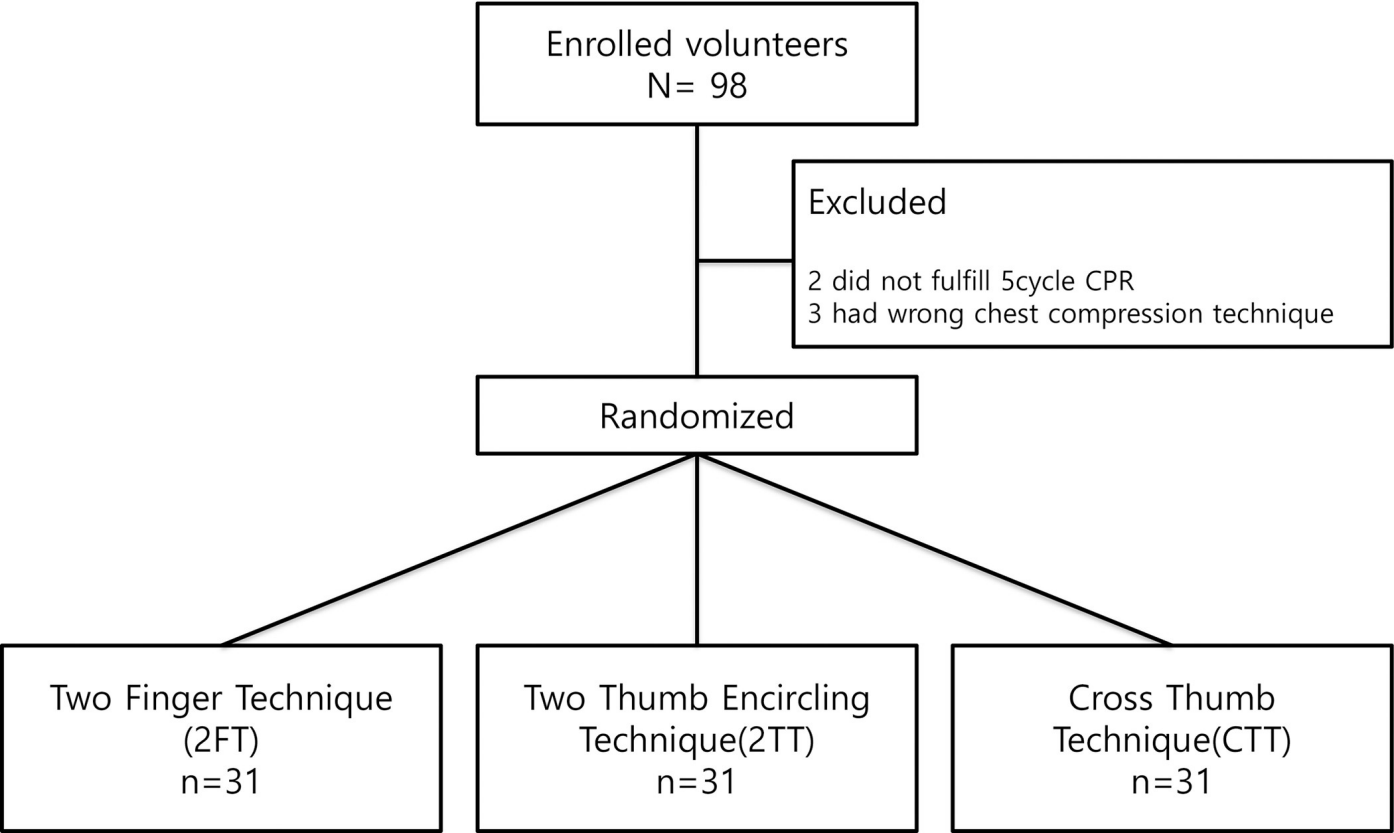
Study outlines.

Ninety-eight volunteers participated in this simulation study. After the BLS lecture video, study instruction, and simulation practice, the volunteers enrolled in this study. We excluded two participants who did not complete five cycles of CPR due to fatigue and pain during the practice time. Three additional participants were excluded because they performed a chest compression technique other than the one to which they had been randomized. We randomly allocated the participants to three groups: 2FT, 2TT, and CTT. Each group included 31 participants, and their CPR performance was recorded (Fig 3).

The median age of enrolled participants was 30.0 (IQR 27.0, 32.0) years. Fifty-seven participants were women (61.3%), and 36 participants were men (39.7%). There were 44 doctors (47.3%), 37 nurses (39.8%), and 12 emergency medical technicians (12.9%) in this study. General characteristics of participants show Table 1. The job, sex and age were not significantly different among three groups.

**Table 1:** General characteristics of participants.

	Compression methods			*P-value*
	2FT	2TT	CTT	
	(n = 31)	(n = 31)	(n = 31)	
Job				0.917
Doctors	16	11	4	
Nurses	12	14	5	
EMT	16	12	3	
Sex, male	15 (48.4)	10 (32.3)	11 (35.5)	0.302
Age, yrs	30.7±4.4	29.5±4.0	29.7±4.0	0.467

Values are expressed as number (%) or mean±standard deviations.

2FT, Two finger technique; 2TT, Two thumb encircling technique; CTT, Cross thumb technique; EMT, Emergency medical technicians.

The depth of chest compression in 2FT, 2TT, and CTT were 40.0 mm (IQR 39.0, 41.0), 42.0 mm (IQR 41.0, 43.0), and 42.0 mm (IQR 41.0, 43.0), respectively (Table 2). The depth of chest compression in 2FT was shallower than that in the other two techniques, 2TT and CTT (*P*<0.05) (Fig 3). After post hoc analysis as a pairwise comparison, there were no statistically significant differences between 2TT and CTT. CCF in 2FT, 2TT, and CTT were 73.9% (IQR 72.2, 75.6), 71.2% (IQR 67.2, 72.2), and 71.3% (IQR 67.7, 74.1), respectively (Table 2). CCF was higher in 2FT than in the other two techniques, 2TT and CTT (P<0.05). After post hoc analysis as a pairwise comparison, there were no statistically significant differences between 2TT and CTT.

**Table 2:** Cardiopulmonary resuscitation performance and subjective feedback (NRS) in three groups.

		2FT	2TT	CTT	*P*-value
		(n = 31)	(n = 31)	(n = 31)	
CPR performance	Compression depth (mm)	40 (39–41) ^a^	42 (41–43)^b^	42 (41–43)^b^	<0.05
	Compression rate (bpm)	106 (104–112)	109 (105–114)	107 (103–114)	0.667
	Chest recoil (%)	100 (99–100)	99 (95–100)	99 (92–100)	0.057
	Adequate ventilation (%)	56 (38–80)	50 (20–70)	63 (38–78)	0.260
	Total simulation time (sec)	116.0 (109–122)^a^	119 (114–123)^a^	122 (118–128)^b^	<0.05
	Hands-off time per cycle (sec)	6 (6–6)^a^	7 (6–8)^b^	7 (6–8)^b^	<0.05
	Chest compression fraction (%)	73.9 (72.2–75.6)^a^	71.2 (67.2–72.2)^b^	71.3 (67.7–74.1)^b^	<0.05
	Correct location (%)	99 (86–97)	100 (97–100)	100 (99–100)	0.058
Questionnaires	Difficulty level	3 (1–5)	3 (2–6)	2 (2–4)	0.554
	Fatigue	7 (6–8) ^a^	5 (3–7) ^b^	4 (2–6) ^b^	<0.05
	Pain	7 (6–8) ^a^	5 (3–6) ^b^	5 (3–6) ^b^	<0.05

The different letters indicate significantly different groups on the basis of the Kruskal-Wallis test with multiple comparisons with the Bonferroni correction method. Data show as median (interquartile range).

2FT, Two finger technique; 2TT, Two thumb encircling technique; CTT, Cross thumb technique; CPR, Cardiopulmonary resuscitation; bpm, Beat per minutes; NRS, Numeric rating scale.

Chest compression rates in 2FT, 2TT, and CTT were 106.0 bpm (IQR 104.0, 116.0), 109.0 bpm (IQR 105.0, 114.0), and 107.0 bpm (IQR 103.0, 114.0), respectively (Table 2). Chest recoil in 2FT, 2TT, and CTT were 100.0% (IQR 99.0, 100.0), 99.0% (IQR 95.0, 100.0), and 99.0% (IQR 92.0, 100.0), respectively (Table 2). Adequate ventilation in 2FT, 2TT, and CTT was 56.0% (IQR 38.0, 80.0), 50.0% (IQR 20.0, 70.0), and 63.0% (IQR 38.0, 78.0), respectively. Adequate ventilation in CTT was higher than in 2FT and 2TT, but there were no significant differences (Table 2). Correct location in 2FT, 2TT and CTT were 99.0.0% (IQR 86.0, 100.0), 100.0% (IQR 97.0, 100.0), and 100.0% (IQR 99.0, 100.0), respectively (Table 2). Correct location in CTT was higher than that in 2FT, but there were no significant differences.

There was no statistically significant difference in the difficulty reported by participants in each of the three groups (*P*>0.05). The median NRS scores for fatigue in 2FT, 2TT, and CTT were 7.00 (IQR 6.0, 80), 5.0 (IQR 3.0, 7.0), and 4.0 (IQR 2.0, 6.0), respectively (Table 2). The median of NRS for pain in 2FT, 2TT, and CTT were 7.00 (IQR 6.0, 80), 5.0(IQR 3.0, 6.0), and 5.0 (IQR 3.0, 6.0), respectively (Table 2). The 2FT group showed higher scores in fatigue and pain NRS than the 2TT and CTT groups (*P*<0.05, *P*<0.05, respectively). After hoc analysis as a pairwise comparison, there were no statistical differences in fatigue and pain NRS between the 2TT and CTT groups.

## Discussion

Several attempts and studies have been made to find effective methods that provide high-quality CPR when a single rescuer performs infant CPR. In a recent systematic review and meta-analysis, 2TT was a method that provided high-quality chest compression without compromising ventilation in single rescuer infant CPR [13, 14]. Herein, the depth of chest compression was higher in 2TT and CTT compared with 2FT. Additionally, the compression depth of CTT was comparable to that of 2TT.

However, we thought that the previous studies overlooked the importance of CCF or hands-off time in CPR performance [13, 14]. These studies focused only on adequate chest compression and ventilation volume rather than hands-off time and CCF. Another study reported that the total hands-off time by 2FT and 2TT differ significantly up to 2 seconds [15]. This result might be caused by the location of a single rescuer in the CPR scene. In 2FT, a single rescuer should be located at the shoulder level of the infant to provide chest compression. In contrast, in 2TT, a single rescuer should be located at the hip level of the CPR infant to provide chest compression. Considering that a rescuer in 2FT may be closer to the head of the infant, the difference in single rescuer location in 2FT and 2TT may cause longer hands-off time and lower CCF in 2TT.

While the CCF of 2FT was higher than other techniques, the CCF of 2TT and CTT were not different in our study. The major difference between 2TT and CTT in a single rescuer is the location from which the rescuer performs chest compressions. The 2TT should move from the hip to the face of the infant to provide ventilation after one cycle of chest compression. As 2FT is performed, a rescuer using CTT stands next to the infant’s shoulder and chest, allowing adequate ventilation without moving after one cycle of chest compression. The rescuer can perform adequate ventilation as soon as possible. Therefore, it is supposed that CTT was better than 2TT and comparable to 2FT in CCF, reducing hands-off time without movement. However, the CCF of 2FT was higher than the other two techniques. Previous studies reported similar results that the hand-off time of the 2TT was significantly longer than in the 2FT [16, 17]. Therefore, our hypothesis predicted that the CCF of CTT would be similar to 2FT. However, as a result of this study, the reason why the CCF of CTT was lower than 2FT may be that participants were familiar with 2FT in infant CPR and had shorter training and practice time on CTT. We may achieve a higher CCF by strengthening education in the new chest compression method, CTT.

A rescuer performing 2FT had the advantage of using only one hand and placing it at the side of the CPR infant. It is helpful for rapid changes between chest compression and mouth-to-mouth ventilation and can reduce hands-off time. Recent manikin studies suggest that the 2TT may be associated with lower CCF [15] and incomplete chest recoil especially when performed by single rescuers [11, 18]. For this reason, the American Heart Association (AHA) resuscitation guidelines recommended that the 2FT be used for performing chest compressions in an infant when a single rescuer is performing cardiopulmonary resuscitation. However, it can cause fatigue and pain in two fingers performing chest compression, and weak and slow chest compression can occur in infant CPR. Therefore, when a single rescuer performs an ongoing CPR, the fatigue and finger pain of the rescuer are important factors in maintaining good CPR performance. Our results showed that 2TT and CTT were associated with less pain and fatigue than 2FT in the participants’ questionnaires.

Our study has several limitations. First, we used the infant CPR manikin model, which may not accurately represent real patients. Although the Laerdal manikin and SkillReporter have been used to simulate several CPR performance studies, because we tried to evaluate a new chest compression technique, further studies to confirm the findings of this study and calculate perfusion pressure of human subjects are needed. Second, despite the fact that crossover design is preferred in simulation studies for statistical power, our study was conducted using a randomized control design. We suppose that a crossover design can create performance and detection bias because the aim of our study was to compare the new and original methods, and participants are likely to prefer one method over the other. Third, the simulation study was ongoing for only 2 minutes because it was assumed that the time of single rescuer CPR was usually 2 minutes. Since it was a simulation study, the Hawthorne effect may have appeared. Therefore, all participants may have tried conducting chest compression harder than in the real situation, and indicators related to chest compression may have been good. The test time was short, so the trial results might look better. If a participant performs chest compression for a longer period of time, the results of each compression technique may differ. Moreover, comparing the first and last cycles by extending the time to perform chest compression, the quality of chest compression may vary owing to the pain or fatigue of the rescuer. Further studies are required to verify these limitations.

## Conclusion

Participants using CTT showed a lower CCF than those using 2FT, but performed as well as 2TT at chest compression depth and may help maintain an accurate chest compression position in CPR. Also, rescuers suffered less pain and fatigue performing with CTT. Therefore, a new chest compression technique, CTT may be an effective alternative method for infant cardiac arrest by a single rescuer.

## Supporting information

S1 Data (XLSX)
